# Supplementary material for: Occurrence, Trends, Management and Outcomes of Patients Hospitalized with Clinically Suspected Myocarditis—Ten-Year Perspectives from the MYO-PL Nationwide Database
Source: J Clin Med. 2021 Oct 12;10(20):4672. doi: 10.3390/jcm10204672 (PMC8539273; doi:10.3390/jcm10204672)
Supplement: Supplementary file 1 [file jcm-10-04672-s001.zip › jcm-1357396-supplementary.pdf]

| Procedure/ disease | International Classification of Diseases and Related Health Problems, 10th Revision (ICD-10); ICD 9th Revision, Clinical Modification (ICD-9-CM); NHF billing codes |
|---|---|
| Echocardiography | ICD-9-CM: 37.281, 88.721 |
| Endomyocardial biopsy | ICD-9-CM: 37.25 |
| Invasive or computed tomography coronary angiography | ICD-9-CM: 88.380, 88.55, 88.56, 88.57, 00.66, 00.661, 00.662, 00.668, 36.0, 36.06, 36.07, 36.09, NHF billing code: 5.03.00.0000085 |
| Cardiac magnetic resonance | ICD-9-CM: 88.92, 88.93, 88.94, 88.95, 88.96 (with extensions); NHF billing code: 5.03.00.0000084, 5.03.00.0000124, 5.03.00.0000125 |
| Cardiac catheterization | ICD-9-CM: 37.21, 37.22, 37.23 |
| Heart transplantation | NHF billing code: 5.54.01.0000006, 5.54.01.0000067, ICD-9-CM: 37.5, 37.51, 37.52, 37.521, 37.522 |
| Myocarditis | ICD-10: I40, I40.0, I40.1, I40.8, I40.9, I41, I41.0, I41.1, I41.2, I41.8, I51.4, B33.2 |
| Pericarditis | ICD-10: I01.0, I09.2, I30, I30.0, I30.1, I30.8, I30.9, I31.0, I31.1, I31.3, I31.9, I32, I32.0, I32.1, I32.8 |
| Atrial fibrillation or atrial flutter | ICD-10: I48 |
| Myocardial infarction | ICD-10: I21, I21.0, I21.0, I21.1, I21.2, I21.3, I21.4, I21.9 |
| Ischemic heart disease | ICD-10: I20, I20.0, I20.8, I20.9 |
| Chronic ischemic heart disease | ICD-10: I25, I25.0, I25.1, I25.2, I25.6 |
| Ischemic cardiomyopathy | ICD-10: I25.5 |
| Cardiomyopathy | ICD-10: I42 |
| Dilated cardiomyopathy | ICD-10: I42.0 |
| Pulmonary embolism | ICD-10: I26, I26.0, I26.9 |
| Atrioventricular block | ICD-10: I44, I44.0, I44.1, I44.2, I44.3, I44.4, I44.5, I44.6, I44.7 |
| Other conduction disturbances | ICD-10: I45, I45.0, I45.1, I45.2, I45.3, I45.4, I45.5 |
| Cardiac arrest | ICD-10: I46, I46.0, I46.1, I46.9 |
| Other heart rhythm abnormalities | ICD-10: I49 |
| Ventricular fibrillation or flutter | ICD-10: I49.0 |
| Premature atrial excitation | ICD-10: I49.1 |
| Premature ventricular excitation | ICD-10: I49.3 |
| Sick sinus syndrome | ICD-10: I49.5 |

| | |
|---|---|
| Bradycardia | ICD-10: R00.1 |
| Abnormal heart rhythm tachycardia palpitations | ICD-10: R00, R00.0, R00.2, R00.8 |
| Syncope | ICD-10: R55 |
| Heart Failure | ICD-10: I50, I50.0, I50.1, I50.9 |
| Cardiogenic shock | ICD-10: R57.0, R57 |
| Paroxysmal tachycardia | ICD-10: I47, I47.0, I47.1 |
| Ventricular tachycardia | ICD-10: I47.2 |
| Primary arterial hypertension | ICD-10: I10, I11, I11.0, I11.9 |
| Insulin-dependent diabetes | ICD-10: E10 |
| Non-insulin dependent diabetes | ICD-10: E11 |
| Ischemic stroke | ICD-10: I63, I63.0, I63.1, I63.2, I63.3, I63.4, I63.5, I63.6, I63.8, I63.9, I64, I69.3, I69.4 |
| Transient ischemic attack | ICD-10: G45, G45.3, G45.4, G45.6, G45.9 |
| Embolism and arterial thrombosis | ICD-10: I74, I74.0, I74.1, I74.2, I74.3, I74.4, I74.5, I74.8, I74.9, I63.0, I63.0, I63.1, I63.2, I65.1, I65.2, I63.3, I63.4, I63.5, I66.9, K55.0, I63.0, I63.1, I63.2, I65.9, N28.0, I65.0 |
| Autoimmune diseases | ICD-10: M05, M05.0, M05.1, M05.2, M05.3, M05.8, M05.9, M06, M06.1, M06.2, M06.3, M06.4, M06.8, M06.9, M08, M08.0, M08.1, M08.2, M08.3, M08.4, M08.8, M08.9, M09, M09.0, M09.1, M09.2, M09.8, M30, M30.0, M30.1, M30.2, M30.3, M30.8, M31, M31.0, M31.1, M31.2, M31.3, M31.4, M31.5, M31.6, M31.7, M31.8, M31.9, M32, M32.0, M32.1, M32.8, M32.9, M33, M33.0, M33.1, M33.2, M33.9, M34, M34.0, M34.1, M34.2, M34.8, M34.9, M35, M35.0, M35.1, M35.2, M35.3, M35.4, M35.5, M35.6, M35.7, M35.8, M35.9, M36, M36.0, M36.1, M36.2, M36.3, M36.4, M36.8, M45 |
| Acute kidney injury | ICD-10: N17, N17.0, N17.1, N17.2, N17.8, N17.9 |
| Chronic kidney injury | ICD-10: N18, N18.0, N18.8, N18.9 |
| Chronic kidney disease | ICD-10: N17, N18, N19 |
| Infectious disease | |
| Otolaryngologic and eye | ICD-10: A18.6, H62.0, H66, H66.0, H66.4, H66.9, H67.0, J02.0, J03.0, J36, H60.0, B00.2, B05.3, B08.4, B08.5, B30, B30.0, B30.1, B30.2, B30.3, B30.8, B30.9, H19.1, H62.1, H67.1, J05, J05.0, B02.3, H19.0, H19.2, H60.8, H60.9, H62, H62.3, H65.0, H65.1, H68, H68.0, H70, H70.0, H70.1, H70.8, H70.9, H60, H60.1, H60.2, H60.3, H75.0, H94.0, J01, J01.0, J01.1, J01.2, J01.3, J01.4, J01.8, J01.9, J02, J02.9, J03, J03.9, J04, J04.0, J04.1, J04.2, J06, J06.0, J06.8, J06.9, J40, H13.1, H67.8, J00, J02.8, J03.8, J05.1, H92, H92.0, H92.1 |
| Central nervous system | ICD-10: B90.0, G00.0, G00.1, G00.2, G00.3, G00.8, G00.9, G01, G04.2, G05.0, A84, A84.1, A84.8, A84.9, A85, A87.2, A85.0, A85.1, A85.2, A85.8, A86, A87, A87.0, A87.1, A87.8, A87.9, A88, A88.0, |

| | |
|---|---|
| | A88.8, A89, B00.3, B00.4, B01.0, B01.1, B02.0, B02.1, B02.2, B05, B05.0, B05.1, B06.0, B22.0, B26.1, B26.2, G02.0, G05.1, A87.2 |
| Respiratory | ICD-10: A15, A15.0, A15.1, A15.2, A15.3, A15.4, A15.5, A15.6, A15.7, A15.8, A15.9, A16, A16.0, A16.1, A16.2, A16.3, A16.4, A16.5, A16.7, A16.8, A16.9, A36.0, A36.1, A36.2, A37, A37.0, A37.1, A37.8, A37.9, A43.0, A54.5, A56.4, A69.1, B90.9, J13, J14, J15, J15.0, J15.1, J15.2, J15.3, J15.4, J15.5, J15.6, J15.7, J15.8, J15.9, J16.0, J17.0, J20.0, J20.1, J20.2, J85, J85.0, J85.1, J86, J86.0, J86.9, B01.2, B05.2, B22.1, B25.0, J09, J10, J10.0, J10.1, J10.8, J11, J11.0, J11.1, J11.8, J12, J12.0, J12.1, J12.2, J12.8, J12.9, J17.1, J20.3, J20.4, J20.5, J20.6, J20.7, J21.0, J16, J16.8, J17, J17.8, J18, J18.0, J18.1, J18.2, J18.8, J18.9, J20, J21, J22, J20.8, J20.9, J21.8, J21.9, R05, R06.7, R07, R07.0 |
| Digestive | ICD-10: A00.0, A00.1, A00.9, A01, A01.0, A01.1, A01.2, A01.3, A01.4, A02, A02.0, A02.1, A02.2, A02.8, A02.9, A03, A03.0, A03.1, A03.2, A03.3, A03.8, A03.9, A04, A04.0, A04.1, A04.2, A04.3, A04.4, A04.5, A04.6, A04.7, A04.8, A04.9, A05.0, A05.1, A05.2, A05.3, A05.4, A05.8, A05.9, K67.0, K67.1, A54.8, K67.2, K67.3, A18.3, K67.8, A08, J09, J10.8, J11.8, A08.0, A08.1, A08.2, A08.3, A08.4, A08.5, B05.4, B15, B15.0, B15.9, B16, B16.0, B16.1, B16.2, B16.9, B17, B17.0, B17.1, B17.2, B17.8, B18, B18.0, B18.1, B18.2, B18.8, B18.9, B19, B19.0, B19.9, B25.1, B25.2, B26.3, B94.2, K77.0, K67, K12.2, R10, R10.1, R10.2, R10.3, R10.4, R11, R12 |
| Urogenital | ICD-10: A18.1, A54.0, A54.2, A56.0, A56.2, B90.1, N11.8, N11.9, N13.6, N15.1, N16.0, N34.0, A60.0, A60.1, A60.9, N05, N08, N08.0, N10, N11, N12, N16, N34.1, N34.2, N37.0, N39.0, N41.0, N41.2, N41.3, N41.8, N41.9, A56.1, N34, N73.9, N74, N74.0, N74.1, N74.2, N74.3, N74.4, N74.8, R30, R30.0, R30.1, R30.9, R31, R36 |
| Sepsis | ICD-10: A39, A39.0, A39.1, A39.2, A39.3, A39.4, A39.5, A39.8, A39.9, A40, A40.0, A40.1, A40.2, A40.3, A40.8, A40.9, A41, A41.0, A41.1, A41.2, A41.3, A41.4, A41.5, A41.9, A41.8 |
| Other | ICD-10: A48.1, A48.2, A48.3, A48.8, A49, A49.0, A49.1, A49.2, A49.3, A49.8, A79, A79.0, A79.1, B95, B95.0, B95.1, B95.2, B95.3, B95.4, B95.5, B95.6, B95.7, B95.8, B96, B96.0, B96.1, B96.2, B96.3, B96.4, B96.5, B96.6, B96.7, B96.8, A69.2, A69.8, A69.9, B00, B00.0, B00.1, B00.5, B00.7, B00.8, B00.9, B01.8, B01.9, B02, B02.7, B02.8, B02.9, B05.8, B05.9, B06, B06.8, B06.9, B08.1, B08.2, B08.3, B08.8, B09, B20, B23.0, B20.0, B20.1, B20.2, B20.3, B20.4, B20.5, B20.6, B20.7, B20.8, B20.9, B21, B21.0, B21.1, B21.2, B21.3, B21.7, B21.8, B21.9, B22, B22.2, B22.7, B25, B25.8, B25.9, B26, B26.0, B26.8, B26.9, B27, B27.0, B27.1, B27.8, B27.9, B33, B33.0, B33.1, B33.3, B34, B34.0, B34.1, B34.2, U04.9, B34.3, B34.4, B34.8, B34.9, B94.8, B97, B97.0, B97.1, B97.2, B97.3, B97.4, B97.5, B97.6, B97.7, B97.8, N71, N71.0, N71.1, N71.9, N72, N73, N73.0, N73.1, N73.2, N76.0, N76.2, N77.0, N77.1, R21, R59, R50, R50.8, R50.9, R53, R55, R59.0, R59.1 |
